# Integrated self-management support provided by primary care nurses to persons with chronic diseases and common mental disorders: a qualitative study

**DOI:** 10.1186/s12875-024-02464-8

**Published:** 2024-06-12

**Authors:** Jérémie Beaudin, Maud-Christine Chouinard, Émilie Hudon, Catherine Hudon

**Affiliations:** 1 Faculté de Médecine et des Sciences de la Santé, Université de Sherbrooke, 3001, 12e Avenue Nord, Sherbrooke, Québec J1H 5N4 Canada; 2 Module des sciences infirmières, Université du Québec à Chicoutimi, 555 Bd de l’Université, Chicoutimi, Québec G7H 2B1 Canada; 3 Faculté des sciences infirmières, Université de Montréal, Pavillon Marguerite-d’Youville, C.P. 6128 succ. Centre-ville, Montréal, Québec Canada H3C 3J7

**Keywords:** Self-management support, Primary care, Nursing, Integrated care, Clinical integration, Chronic conditions

## Abstract

**Background:**

More and more people suffer from concomitant chronic physical diseases and common mental disorders, calling for integrated self-management support in primary care. However, self-management support of chronic physical diseases and common mental disorders is not clearly operationalized by guidelines and is still conducted in silos by primary care nurses, especially in favour of chronic diseases. This study aims to better understand primary care nurses’ experience of integrated self-management support for people with physical chronic diseases and common mental disorders.

**Methods:**

An interpretive descriptive qualitative approach was conducted with 23 primary care nurses from family medicine groups in Quebec (Canada). They were selected through purposive and snowball sampling methods to participate in an individual interview. Data were analysed using an iterative inductive and deductive analysis (Rainbow Model of Integrated Care and the Practical Reviews in Self-Management Support (PRISMS) taxonomy).

**Results:**

Nurses’ experience of integrated self-management support for people with CD and CMD was structured around: (1) elements of the approach; (2) clinical integration through prevention and health promotion; and (3) operationalization of integrated self-management support. Several elements deemed essential to integrated self-management support were identified. Nurses offered integrated self-management support through prevention of risk factors and promotion of a healthy lifestyle for physical chronic diseases and common mental disorders. Nurses’ self-management support activities included education, action plans, monitoring, and many practical, psychological, and social support strategies. A model of integrated self-management support for primary care nursing is proposed to better understand its clinical integration.

**Conclusion:**

This study presents clinical integration of self-management support and activities for people with physical chronic diseases and common mental disorders in primary care settings. Understanding integrated self-management support will help implement future interventions.

**Supplementary Information:**

The online version contains supplementary material available at 10.1186/s12875-024-02464-8.

## Background

More and more people suffer from concomitant chronic physical diseases (CD) and common mental disorders (CMD) and this is recognized as a major health issue [1, 2]. As defined by the National Institute of Health and Care Excellence (NICE) guidelines, common mental disorders include depression, generalized anxiety disorder, panic disorder, obsessive-compulsive disorder, post-traumatic stress disorder, and social anxiety disorder [3]. Among these conditions, depression and anxiety disorders are the most common in primary care [4]. CD and CMD are responsible for most deaths and morbidity worldwide and their prevalence is steadily increasing. Combined, these conditions have a negative impact on health in terms of morbidity [5] and mortality [6]. The bidirectional relationship between CD and CMD exacerbates the challenges within the healthcare system, making this population more likely to receive fragmented care [7, 8].

Primary care is recognized as the cornerstone for prevention and health promotion, as well as for the management of CD and CMD [9]. Primary care nurses play an important role in the detection, prevention, and management of CD and CMD [10, 11] through holistic assessment of the person, health promotion, care management, nurse-family physician collaboration, and service planning [12]. Self-management support and health education are among nurses’ primary activities [13, 14]. Self-management of CD and CMD, as well as self-management support, are highly recommended by the current guidelines [15–17].

Health professionals offer self-management support to help the person manage their health using a variety of activities, such as problem-solving and goal setting as well as many support strategies to manage medication and health habits [18]. There are multiple benefits to self-management support, namely, improvement of biological markers (blood pressure, cholesterol), self-efficacy, and quality of life, as well as a decrease in depressive and anxious symptomatology and relapse [19–21]. Integrated self-management support for CD and CMD is proposed as a potential solution to improve health outcomes for this population [22–24]. However, current guidelines for the management of CD and CMD (e.g., NICE guidelines [15]) are not clear on the clinical integration of self-management support for this population, or with respect to its components [23]. Self-management support of CD and CMD is still conducted in silos, especially in favour of CD [25].

A scoping review of integrated self-management support interventions for CD and CMD by primary care nurses clarified its main characteristics [24]. Using the Rainbow Model of Integrated Care by Valentijn et al. [26], this review defines clinical integration of self-management support as “coordination of person-focused care for a complex need at stake in a single process across time, place and discipline.” Four main attributes of integrated self-management support were proposed: person-focused self-management support (holistic; based on the person’s needs, priorities, preparation, knowledge, and health literacy); co-created self-management support (personalized; egalitarian nurse-person relationship); shared responsibility and joint agreement on the self-management support (tailored follow-up; common agreement; individualized and shared action plan); and person-coordinated self-management support [24]. Integrated self-management support interventions encompassed activities such as therapeutic education, problem-solving, goal setting, support for medication management and self-care, among others aimed at promoting healthy behaviours and managing both CD and CMD [24].

Studies on integrated self-management support interventions showed improved health outcomes, such as better haemoglobin levels, lower depression scores, lower blood pressure, greater satisfaction with care across conditions (CD and CMD) [27–31]. However, there seems to be a lack of in-depth description of integrated self-management support activities and interventions, as well as a paucity of qualitative studies, none of these studies having evaluated the real-life practice of integrated self-management support by primary care nurses [24].

## Methods

### Aim

The aim of this study was to better understand primary care nurses’ experience of integrated self-management support for persons with CD and CMD, specifically regarding clinical integration and the activities they carried out.

### Design

An interpretive descriptive qualitative study was conducted using Thorne’s approach [32]. Considered as pragmatic, this approach helps answer clinical questions, resolve problems, and propose a theoretical framework to explain the results [32].

### Sampling, setting, recruitment (criteria) and procedure

The target population included all nurses in family medicine groups (FMGs) and university family medicine groups (FMG-Us) across the province of Quebec (Canada) who were interested in sharing their experience of self-management support. In Quebec, FMGs are primary care medical clinics where family doctors practise in collaboration with other professionals, including nurses [33]. FMG-Us differ from FMGs in that they are university-affiliated learning environments for medical residents and other health trainees [34]. Two sampling strategies were used for recruitment: purposive sampling, based on inclusion criteria, and snowball sampling, where people who had already been recruited or approached helped recruit others [35].

Several means of recruitment were used. First, participants were recruited directly from FMGs in Quebec’s Saguenay-Lac-Saint-Jean (SLSJ) and Estrie administrative regions. These are urban regions and include two of the largest municipalities in the province (Saguenay and Sherbrooke) [36]. Two other organizations were approached: The Quebec FMG nurses’ virtual community of practice (CoP) and the Quebec Order of Nurses (QON). The CoP primarily includes nurses from Quebec FMGs. Invitations were emailed to these nurses, either through an intermediary (for CoP and FMG candidates in the Estrie and SLSJ) or directly by the principal investigator (for QON candidates).

To be included in the study, nurses had to: (1) have at least one year of FMG experience; (2) have completed at least one follow-up (within the last year or still active) with people who had at least one CD (any physical CD) and one CMD (e.g., depressive and anxiety disorders); and (3) speak French. Recruitment took place over a one-year period (January 2022 to January 2023). Seven nurses were excluded for various reasons, namely lack of experience with people with CMD (*n* = 3); lack of response during recruitment (*n* = 3); and one person withdrew during the data collection phase. Recruitment continued until the phenomenon had been sufficiently explored and redundancy in collected data was observed [37, 38]. Twenty-three nurses from several FMGs across Quebec participated in the study.

### Data collection and analysis

Data collection and analysis were conducted iteratively from January 2022 to February 2023 [32, 39]. Data collection was performed using semi-structured individual interviews and a socio-demographic questionnaire; and reflective and methodological journals were used for data analysis [32, 40, 41]. The use of semi-structured individual interviews helped to circumscribe the study phenomenon in depth while allowing participants to express themselves freely [41]. An open-ended interview guide was developed based on the research questions, including follow-up questions inspired by the Integrated Self-Management Support Framework [24] and Valentijn et al.’s conceptual model [26, 42] (see Additional file 1).

The guide was tested prior to the first interviews with two FMG nurses who had experience in the management of CD and CMD, in order for the interviewer to become familiar with the questions and ensure fluidity [40]. The guide was refined as interviews progressed to further investigate underdeveloped themes [43]. Interviews were conducted virtually, using the Microsoft Teams platform, by the first author, who is trained in qualitative research. They were recorded directly via this platform and transcribed verbatim.

Data analysis was conducted using Miles et al.’s [39] inductive and deductive thematic analysis method. This pragmatic analysis method is consistent with Thorne’s approach [32] as it enables inductive in-depth exploration of a phenomenon, while drawing inspiration from existing theories [39]. Briefly, the analysis involved three activities: data condensation, data display, and drawing conclusions and verification, which took place simultaneously throughout the research process. Essentially, they were carried out after each interview until an adequate description of the phenomenon was obtained. Through an inductive process employing codes, memos and annotations, data condensation was conducted (first and second coding cycles) during multiple read-throughs. Memos and annotations were used to clarify and reflect on interpretation of emergent themes and subthemes. In addition to inductive coding, an initial list of deductive codes were drawn up using two models: (1) the Integrated Self-Management Support Framework [24] for the modelling of integrated self-management support using the four main attributes of integrated self-management support as previously proposed [24]; and (2) the PRISMS (Practical Reviews in Self-Management Support) taxonomy [44] for the description of self-management support activities. This list of deductive codes was used to organize the coding structure and, over the course of the analysis process, several inductive codes emerged and were merged. The inductive and deductive analysis enabled us to clarify certain previously stated themes of integrated self-management support in greater depth, as well as to explore and integrate new themes. A sample coding tree is presented in Additional file 2. Coding was done by two researchers (JB and EH). Data display consisted of multiple iterations of summary tables and conceptual structures to visualize links and trends between codes and themes, and to interpret data. Finally, drawing conclusions and verification were done through (1) independent co-analysis (JB, EH) involving discussions about the analysis process and themes; and (2) researcher triangulation with co-investigators (MCC, CH) involving several discussions about codes, themes, conceptual structures, and summary tables. Reflexivity was applied throughout all analysis activities.

Thorne’s rigor criteria guided the design and execution of this study. The research process was carried out mainly by the first author, fostering his immersion in the data and the project, and enhancing credibility of the interpretation [32, 45]. Researchers (JB, EH, CH, MCC) paid particular attention to explore every aspect of the findings and to any new issue raised by nurses [39]. Reflexivity was ensured through a methodological transparency by the use of a reflective journal and reflexive memos written throughout the data analysis [46]. The notes recorded in the reflective journal enabled the first author to maintain a certain distance from the data by questioning the research process and results, and by taking his subjectivity into account [39]. Finally, the research process was detailed in a methodological journal and audit trail, making it possible to trace every methodological decision and ensure transparency [39].

The multicentre research project was approved by the CIUSSS de l’Estrie-CHUS Research Ethics Board, and written consent was obtained from all participants.

## Results

### Description of participants

Nurses were recruited from 11 of 18 regions across Quebec. As shown in Table 1, the sample consisted of 23 nurses (22 women and one man), mostly aged between 35 and 54, with 9 to 40 years’ experience in nursing. All participants had at least a bachelor’s degree and the majority worked full-time. In general, nurses were more experienced and trained in CD than in CMD.

**Table 1:** Characteristics of participants

Variables	Statistics
Gender	**Female: 22** Male: 1
Age	24–34 years: 3 **35–44 years: 12** **45–54 years: 7** 55–64 years: 1
Training	**Bachelor’s degree: 22** Master’s degree: 1
Years of experience (total)	**Average: 19 years** Median: 17 years (min: 9; max: 40)
Years of experience (in FMG)	**Average: 9 years** Median: 9 years (min: 1; max: 19)
Work regime	**Full time: 17** Part time: 6
Years working with people with CD in an FMG	**Average: 9 years** Median: 9 years (min: 0; max: 19)
Other than in FMGs, have you ever worked with people with CD?	**Yes: 20** No: 3
Training received for CD self-management support	**Yes: 18** No: 5
Years working with people with CMD in an FMG	**Average: 7 years** Median: 7 years (min: 0; max: 19 years)
Other than in FMGs, have you ever worked with people with mental disorders?	**Yes: 15** No: 8
Training received for CMD self-management support	Yes: 10 **No: 13**

Nurses actively collaborated with several health professionals within the FMGs. The most frequently cited were family physicians, social workers, and pharmacists, as well as many others (nutritionists, psychologists, nurse practitioners, respiratory therapists, physiotherapists, occupational therapists, special educators). Nurses managed the care of a variety of client groups, ranging from paediatric to geriatric follow-up, including perinatal care, women’s health, and cognitive disorders. The main focus was on CD (diabetes, hypertension, dyslipidemia, lung disease). All cases of CMD were comorbid with CD, and except for one nurse, none followed up exclusively on CMD.

### Description of settings and context

Seventeen nurses worked in FMGs and six in FMG-Us. FMGs/FMG-Us varied in size, with smaller FMGs being allocated fewer professional and administrative resources than larger FMGs [33, 34]. Table 2 provides an overview of the settings and distribution of nurses.

**Table 2:** Distribution of nurses by FMG/FMG-U

FMG/FMG-U size	FMGs	FMG-Us
Small	6	0
Medium	5	3
Large	6	3
Total nurses	17	6

### Nurses’ experience of integrated self-management support for people with CD and CMD

Nurses’ experience of integrated self-management support for people with CD and CMD was structured around : (1) elements of the approach; (2) clinical integration through prevention and health promotion as part of a person-focused approach; and (3) operationalization of integrated self-management support.

#### Elements of the approach

Nurses presented an approach consisting of several elements deemed essential to integrated self-management support: a person-focused approach, co-creation of self-management support process and adaptation of integrated self-management support on an autonomy-dependency continuum.

#### A person-focused approach

Nurses’ self-management support approach was holistic and humanistic. Nurses provided care by considering the person before the illness, being a guide for that person, sharing experiences, and treating the person as they would wish to be treated:

“Now, when I see that someone is coming in for the management of a chronic disease of some type, I always use part of our time to talk about stress and all that. When we do [the assessment] of lifestyle habits, I realize, ‘Ah, maybe there’s something there.’ Sometimes, I’ll set aside the teaching about chronic illnesses to assess the person’s mental health a little more. I’ll take my scales, the PHQ-9 or the GAD-7, and I’ll dig into that a bit. […] because it’s probably going to influence their chronic illness or whatever. This is when I see that [my self-management support] becomes much more holistic for everyone in my practice.” (nurse 4)

In the presence of people with CMD, nurses tended to be more sensitive to the person’s psychological state, and to devote more time and resources to them. For all nurses, adapting the self-management support to the person’s current state, progress, needs, desires, and abilities was essential.

#### Co-creation of the self-management support process through active participation and shared responsibility

The relationship was essential to the whole self-management support process. It was a priority for nurses to create a strong bond of trust to develop a prolonged and lasting relationship with the person. However, the development of this relationship varied from one person to another and required the use of different strategies: in-depth understanding of the person; sharing personal experiences; giving importance to lived experience; gradually building the relationship; paying personal attention; personalizing speech; being in the moment; and ensuring a safe environment. For nurses, there were clear benefits to this type of relationship as it facilitates and improves care; strengthens the bond; helps anticipate and reduce disease-related difficulties; facilitates confidences and helps achieve lasting objectives:

“Over the past 5 years, I believe my practice has evolved in this direction, and I tend to use much more ‘tender loving care.’ […]. I used to feel like the more expeditious I was, the more time I’d have, the more patients I’d see. […] Whereas, in the end, when you take a little more time, well, it takes less time. [The person] is more attentive, more collaborative, and then [the person] is happy to see you and have a connection with someone they trust.” (nurse 22)

Furthermore, the adoption of attitudes and behaviours that are conducive to the co-creation of the self-management support process by nurses and the person was raised by many interviewees. It was part of the foundation of the integrated self-management support approach because attitudes and behaviours have a considerable impact on the process and the person. For nurses, this meant being interested, motivated, humble, humanistic, authentic, versatile, open, and transparent.

Once the relationship was established, it was easier to actively involve the person, create a plan together, and share the responsibility for care planning and follow-up. As mentioned above, the person’s involvement was a necessary part of the teamwork. To achieve this, several strategies were raised by the nurses, such as starting from the person’s experience to build the self-management support, letting the person lead the interview and make decisions, asking for the person’s opinion and expectations, ensuring that the choices of support strategies come from both the nurse and the person and are well understood, and involving family and friends or partners:

[Discussing the plan] “For me, it implies that the patient makes decisions about their goals and is involved in the process. I’m not the one who’s going to say, ‘I think it would be better if you took a 30-minute walk every day. It’s going to help your depression and your hypertension.’ You know, I’m not here… If I have someone who says, ‘I don’t have any ideas,’ then I’ll make suggestions. But I want it to come from them, so they choose their objectives. In any case, this is how it becomes more individualized, and I think this is how we achieve success.” (nurse 2)

By letting people establish their own objectives, nurses reinforced the individual’s autonomy in choosing the direction of self-management support and share responsibility with them.

Co-creation of the self-management support was achieved through active participation and a clear sharing of responsibilities on all aspects of the self-management support, including follow-up, choice of treatments, and steps to be taken. Nurses expected people to take charge of their own healthcare as much as possible: To follow up with the nurse when requested or on their own initiative; to make appointments; to take the necessary steps for their health. Nurses respected decision-making autonomy and gave the person enough space to encourage autonomy while providing them with the appropriate tools, depending on the situation. These included screening or assessment tools (e.g., PHQ-9 for depression, GAD-7 for anxiety), teaching tools (e.g., booklets, one-pagers, pamphlets), and medical protocols to support medication adjustment. However, sharing power in the relationship can lead to issues related to safety (if the person is not capable of being autonomous), power (by removing the care-related responsibility from the nurse), dependency (as support is dependent on the partnership between the nurse and person), and performance (where the nurse feels responsible for the person’s results). In all cases, nurses viewed the sharing of responsibilities positively and relied on adapted access to care, based on the person’s needs, to better meet them. They practised self-management support in the spirit of collaboration and partnership:

“In the relationship, I like to share the power with my patient. I’m more interested in collaboration.” (nurse 8)

Continuity of care and services, including interprofessional collaboration and relational continuity was an important part of the co-creation of the self-management support process. From the nurses’ perspective, the self-management support they provided specifically for CMD was not as fully developed as what they achieve in physical health. For several reasons, including lack of knowledge and skills, nurses tended to refer to social workers, psychologists, or family physicians for more comprehensive care. When dealing with mental health issues, some were more likely to play a pivotal role within the interdisciplinary team, especially in larger FMGs where more professional resources are available.

“I continue to manage their care, but I refer them. […] For example, I call the social worker, and then I say, ‘[[Social worker], come into my office, I’d like to introduce you to someone.’ […] I know my patient. So, I introduce the person and then I choose which professional would be most appropriate for that person.” (nurse 12)]

In the presence of a person with CMD, nurses emphasized the importance of relational continuity throughout the liaison process with another professional, even after they referred the person.

#### Adaptation of integrated self-management support on an autonomy-dependency continuum

As a person’s autonomy fluctuates according to their condition and abilities, nurses adapted self-management support along a continuum from dependency to autonomy to better align with the reality and complexity of the person’s situation. As much as possible, the nurses tried to empower people, while always aiming to prevent the situation from deteriorating or becoming too difficult for the person to manage.

“[…] generally, we assess their level of motivation to take care of themselves. Then, you know, if I have a patient who’s not motivated, who doesn’t feel well and we’ve just started medication or it’s not going well at all and he’s grieving, I’m not going to give him an assignment, you know (laughs). We’re going to adapt to the person who’s in front of us.” (nurse 7)

Tailoring self-management support to the person’s actual condition enabled nurses to avoid ethical issues that might arise from self-management support, as an example if the person lacks the capacity to take care of him/herself and the self-management skills required are complex.

#### Clinical integration through prevention and health promotion

As part of the person-focused approach, integrated self-management support was provided through activities including promotion of a healthy lifestyle, risk factor prevention, and education on disease interaction.

#### Promotion of a healthy lifestyle and prevention of risk factors

The most popular strategies used to ensure that mental and physical health are targeted as part of integrated self-management support were to address lifestyle (e.g., physical activity, diet, sleep) and risk factors including stress, social isolation, and substance use (tobacco, drugs, alcohol) to initiate a discussion about mental health:

“There are plenty of things we can recommend for mental health, which also work for all physical illnesses. Everything to do with lifestyle habits: good sleep hygiene, diet, exercise… […]. So, often, it’s by asking a question that people will open the door. [For example], ‘Do you drink alcohol?’, the patient replies, ‘yes […] It’s because I’m too stressed, after my big days. You know, I need my beer when I get home…’ [The nurse replies], ‘OK, but tell me more about why you need your beer…’ This makes it easier to get in. You know, without asking the at-risk person, ‘How is it going with your mental health?’” (nurse 15)

For nurses, the synergy between physical and mental health, and the impact of a healthy lifestyle and risk factors on health, were well-established concepts. Assessing lifestyle habits and risk factors provided an overall view of the person’s functioning, where, for example, halting a lifestyle habit could mean the deterioration of a person’s condition:

“I’ll ask them, ‘How are you? Are you still taking walks?’ Because I know that walking is good for [the person]’s mental health. So, if they say that they have stopped going for walks, something that tells me that they are not doing so well. ‘Are you still doing your activities?’ I’m going to assess lifestyle habits and if something has changed, it’s going to raise a red flag… You know, when someone is not going for walks any more, who’s not eating as well, I’ll be able to delve deeper into that.” (nurse 24)

The notion of stress management was particularly prevalent among nurses, as it was a precursor to physical and mental health issues, and it was the closest thing to mental health self-management support.

#### Disease interaction education

Subsequently, by probing lifestyle habits and risk factors, it was easy for them to teach people about various topics and explain the interaction between physical and mental illnesses. Nurses often used analogy, metaphor, or comparison to explain this interaction. In addition, teaching was also an opportunity to talk about effective support strategies for both physical and mental health (e.g., stress management, exercise, and others); to discuss medication; to raise awareness of the need to focus on both mental and physical health; and, to debunk certain misconceptions or preconceptions. Education about the interaction between diseases was one of the most concrete strategies for bridging the gap between physical and mental health:

“When I have diabetic patients, I make a connection between blood sugar fluctuations and cortisol levels, for example. So we’re going to marry that to explain to them [that their mental health problem] also has an impact on their physical health […] as I often say to my patients, ‘We’re trying to facilitate calm feelings in your mind, but it’s also going to go through your body.’ I often allude to a machine, you know. A typical example of this is: If you don’t take good care of your machine, it won’t be able to do much mileage.” (nurse 20)

#### Operationalization of integrated self-management support

Several self-management support activities were performed by nurses to operationalize the integrated self-management support approach. Data about how nurses integrated self-management support were condensed into nine activities inspired by the 14 components proposed by the PRISMS taxonomy [44] (see Additional file 3 for details of this condensation). Table 3 presents a summary of the self-management support activities carried out by nurses.

**Table 3:** Self-management support activities

Summary of self-management support activities	Integrated self-management support activities
• Mostly focused on CD, with *less emphasis on CMD* • Subjects: Depending on pathology, pathophysiology, complications, signs and symptoms, medication, treatments • Several teaching tools (*few for CMD*) from various sources (web, nurses) and in various formats (video, paper)	Biopsychosocial therapeutic education
• Both formal and informal action plan (*little emphasis on CMD*) • Problem solving, action planning, and goal setting: person-focused and person-driven; longer term; short and simple goals, SMART (*few goals for CMD or indirect focus*) • Topics: lifestyle habits; medication; substance use	Shared individualized action plan
• Systematic file review (objectives, previous notes, assessments, medication, history of the current disease) • First meeting = comprehensive biopsychosocial assessment • *Follow-up of CMD absent or non-systematic*, but closer follow-up when present	Biopsychosocial monitoring
• Regular support for medication intake and adjustment (*less for CMD)* and for adoption of new behaviours (often related to lifestyle habits)	Adherence support
• Mostly related to lifestyle support, management of CD (diabetes; hypertension) and *little or none for CMD*; and various psychological strategies • *No mention of training and practice in communicating with other professionals*	Support for practical self-management skills
• Education and support related to the adoption of a healthy lifestyle and to risk factors: diet, sleep, physical activity, smoking, weight, substance use (drugs, alcohol), stress	Lifestyle support
• Diversified strategies, *but sometimes not very elaborate, relying on feeling or instinct* • Strategies used: o Cognitive: communication technique (active listening, counselling, reflection, silence, reformulation, non-verbal), normalization, reframing, de-dramatization; o Emotional and experiential: stress and emotional management; o Behavioural: motivational interviewing, follow-up diary; o Psychoeducational: reading, writing	Psychological strategies
• Education on available resources (other professionals, community organizations, phone applications, employee assistance programme) • Liaison with internal (social worker or psychologist) or external resources (community organizations) • Emergency management and safety net	Social support and resource liaison
• Access by telephone (direct line; fixed time) • Leaves contact information for self and other resources as needed • Dedicated time slots for one-time and urgent requests	Self-management support accessibility measures

CD: Chronic disease; CMD: Common mental disorders

Italics: elements with gaps in clinical integration

A variety of CD-related self-management support activities were carried out and the nurses argued that they were competent in this field. Establishing a concerted action plan with the person was often the starting point for adapting and co-creating the self-management support. Nurses implemented a variety of interventions that were tailored to the person, and applicable to both CD and CMD, such as biopsychosocial education; self-management skills support, psychological strategies, lifestyle habits and risk factors; and social support. Biopsychosocial monitoring, as well as support for medication and behaviour adherence were also implemented. The self-management support process was adapted across an entire autonomy-dependency continuum. While not exclusive to CMD, certain activities were more likely to be applied by nurses in specific CMD contexts, such as psychological strategies (e.g., communication technique, stress and emotional management), social support and resource liaison, and strategies to access increased self-management support.

One reported aspect was the broad generalist role played by nurses, often giving minimal priority to CMD in the context of a specific follow-up. As shown in Table 3 (underlined), some activities demonstrated a more summary approach to CMD and nurses sometimes felt it preferable to refer the person to another health professional. Based on the person’s needs, nurses implemented intervention strategies, often psychological, to ensure the integration of mental health. A wide range of psychological interventions were described by the nurses. Here again, support for physical CD was evident and clearer, as guidelines are often already in place, as well as a definition of the nurse’s role in this regard, compared to guidelines for CMD, which are less available in these settings, and where nurses’ involvement remains unclear.

### Model of integrated self-management support in primary care nursing

**Fig. 1:**
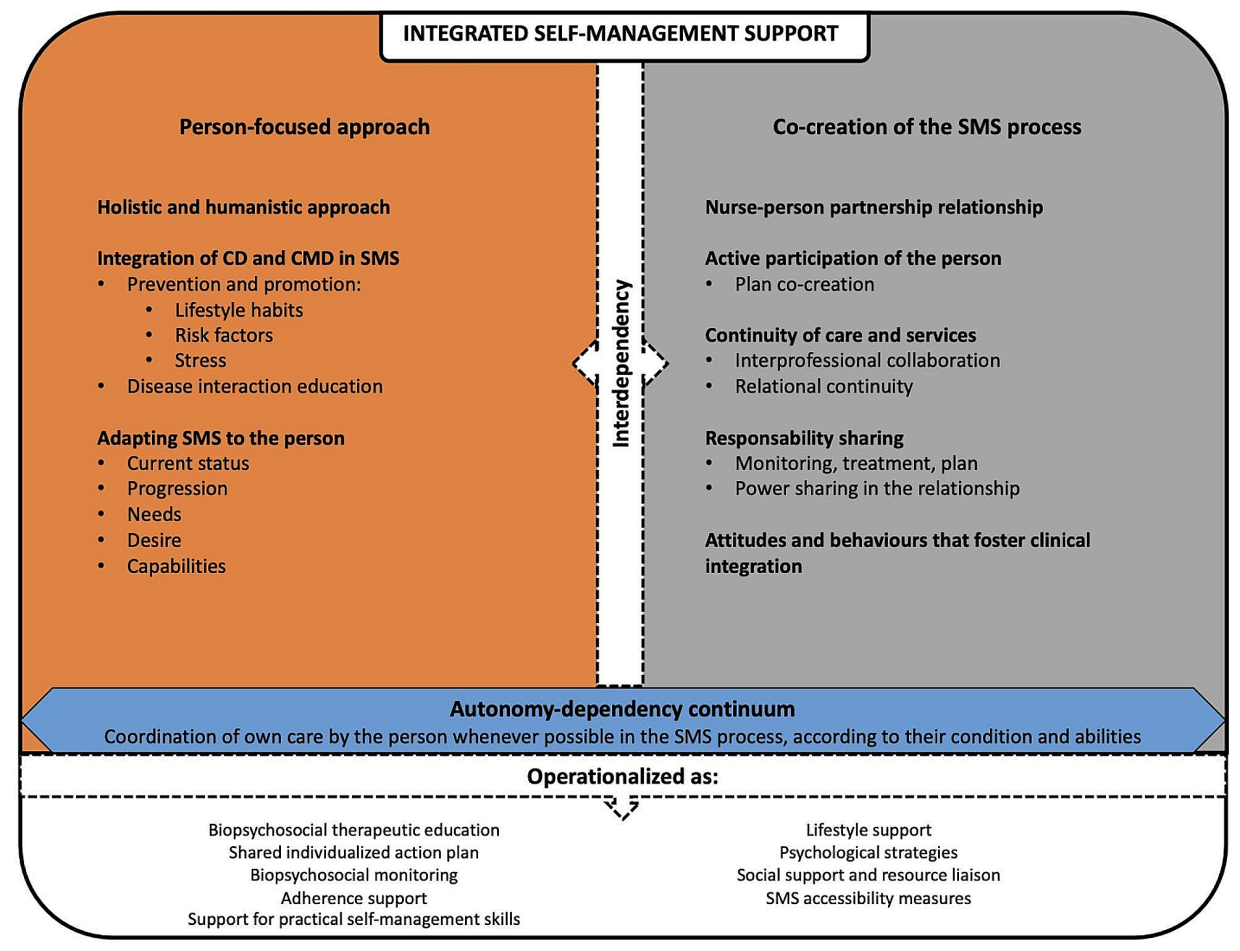
Model of integrated self-management support in primary care nursing

Based on the results of this study, a model of integrated self-management support for CD and CMD in primary care is proposed (Fig. 1). This theoretical model is based on the work of Beaudin et al. [24], as detailed earlier, and goes a step further by adding new elements and links between concepts. First, the new model reduces the number of categories to two: “person-focused care” and “co-creation of the self-management support process.” The original categories (co-creation of the self-management support; shared responsibility and joint agreement on the self-management support; and, coordination by the person) were merged under “co-creation of the self-management support process,” as all these elements are interrelated. As for “interdependence,” according to Valentijn et al. [26], it is necessary to have both a person-focused approach and co-creation of the care process, hence the addition of this element to the framework. In practice, nurses had a similar discourse:

“[…] I tell them, I repeat to them, ‘It’s you and me, we work as a team. I’m nothing without you. By this, I mean that if I don’t have data, I can’t work. I can explain to you what diabetes is. After that, I can’t work without you; I can’t personalize your treatment, you know? I can give you a global treatment, give you a scale and then hope you don’t get retinopathy. But if you work with me, we’re going to have the best of both worlds.’” (nurse 22)

For nurses, it was necessary to adapt the self-management support to the person, while depending entirely on the active participation of the latter to be able to offer truly person-focused support.

## Discussion

This study developed the concept of integrated self-management support for people with CD and CMD by primary care nurses. On the one hand, clinical integration of self-management support for CD and CMD was studied based on nurses’ experience and the literature. Next, the activities practised by nurses were clarified, and some strengths and gaps related to practice in specific mental health contexts were highlighted. Finally, a model was proposed to synthesize the attributes of this approach.

When comparing the nurses’ experiences in this study with the scoping review carried out to identify integrated self-management support interventions and their characteristics [24], several similarities became apparent. There is still significant emphasis on considering the person holistically and prioritizing the development of a strong relationship with them. This study goes further by explaining how nurses integrate physical and mental health into support activities, situating the level of intervention related to prevention and health promotion; and explaining how nurses apply an integrated self-management support approach.

Many other important points emerged regarding clinical integration of self-management support. The lack of knowledge and skills specific to CMD (anxiety or depression) is a well-known problem in Quebec’s primary care settings [47, 48], and elsewhere in the world [49, 50]. In an integrative review targeting primary care nurses’ knowledge gaps and learning needs in mental health [51], the authors identified several reasons for these gaps: inadequate preparation (initial and continuing training); limited exposure to theory or clinical experience; lack of confidence and motivation; and, the low priority given to mental health. However, studies are clear regarding the positive impact on health (reduction in depressive and anxiety symptoms) and the level of satisfaction of those receiving care from competent primary care nurses trained in mental health [21, 52]. Despite a growing need for more skilled primary care nurses in the field of mental health, the offer of training and clinical support must evolve and be valued more to foster the advancement of nurses’ practice as well as the quality of care provided to the person [9, 25, 51, 53].

When comparing nurses’ practice to current guidelines [13], nurses’ integrated self-management support has many positive points, but could be improved. This is an interesting observation, as it reinforces the need for more CMD training for nurses, so that they understand their interventions, both in terms of the choice of strategies to be applied and their effectiveness. The results of this study lead us to rethink the importance of the approach in the management of CD and CMD for their clinical integration, by concurrently developing the components related to the approach (a partnership relationship; continuity of care; a humanistic and holistic approach; sharing of responsibilities and decisions related to the plan; communication) and the practical skills (support strategies; knowledge and skills). Focusing on approach-related components, an integrative review of non-technical competencies (or soft skills) in nursing [54] defines these as social and cognitive competencies, referring to the caring aspect and to nurses’ tacit and intuitive knowledge. Referring specifically to the self-management support provided as part of primary care nurses’ practice, many activities require non-technical skills on the part of the nurse (e.g., developing relationships, collaborating) [12, 55]. Of these non-technical skills, the ability to communicate well, to work as part of a team (leadership), to demonstrate creativity, sensitivity, empathy, and emotional intelligence are the most frequently cited as important to have and to develop, as these skills have numerous benefits [54, 56, 57]. Thus, it has been recommended that greater importance be given to the development of approach-related skills, i.e., the development of the partnership relationship; person-centred care; and, shared decision-making [58–60].

In terms of integrated self-management support activities, the results of this study demonstrate a wide range of activities and strategies. Several authors have examined the best intervention strategies to apply, but the optimal combination for this clientele remains uncertain [14, 61, 62]. There are promising practices for managing people with CD and CMD, such as partially supported and self-directed digital mental health interventions to monitor and support self-management of chronic conditions [63], care management through collaborative models [28], or different forms of psychotherapy [64, 65]. Among the suggested strategies, guidelines for the treatment of depression concomitantly with CD [15] recommend the use of low-intensity psychosocial interventions prior to pharmacotherapy. These interventions include all components of lifestyle habits (physical activity; sleep hygiene; stress management; substance use and anxiety); active monitoring; and some activities common to self-management support, such as psychological education, problem solving, partnership, and other behaviour change therapies. The effectiveness of psychosocial and behaviour change interventions has been demonstrated in a variety of settings, for both CD and CMD, in both disease-specific and concomitant contexts [16, 17, 64, 66–72]. Given their efficacy and low cost, these interventions are wise choices to develop [73]. More broadly, several authors agree that the development of self-management skills should involve behaviour change therapies [73] and that the use of these therapies within self-management support must be further developed during initial and continuing training [74, 75]. In this regard, it would be promising to develop integrated self-management support within an integrated behavioural health approach in primary care, as it focuses on holistic support (CD and CMD) in primary care of a person using behaviour change therapies. Integrated behavioural health is an emerging primary care approach to managing behavioural health issues (e.g., mental health, substance use, lifestyle habits) of people with CD and CMD, which requires several competencies and skills, including a humanistic approach that takes the whole person into account [76, 77]. This approach is increasingly recognized for the management of CMD in primary care [78, 79]. Nurses, supported by training, are well positioned to practise this approach [80].

Finally, the results enabled us to present a model of integrated self-management support (Fig. 1). This model has many applications, both theoretical (conceptualization and understanding of integrated self-management support) and practical (description and development of self-management support interventions in primary care contexts for people with CD and CMD). Discussed briefly in this study and also reported by other authors [24, 81–83], several important influencing factors can modulate the clinical integration of self-management support. To better understand them, an in-depth study of factors influencing clinical integration and strategies to improve integrated self-management support will be carried out in a future publication.

### Strengths and limitations

To our knowledge, this is the first qualitative study to explore the experience of primary care nurses with integrated self-management support for CD and CMD. The study was carried out in accordance with Thorne’s rigorous criteria [32]. A triangulation of the results between researchers and with the literature was carried out; a diversification of experiences was obtained (several sites and regions of Quebec); and, data saturation was obtained after 20 interviews [39, 84]. The methods used and the clinical research questions were consistent with Thorne’s approach [32]. A rich in-depth description of the results, supported by verbatim quotes and taking into account information from all facets, was produced [39, 84]. A clear and detailed description of the process was achieved by keeping a methodological journal. Peer debriefing was conducted to discuss the analysis process and results [39, 84, 85].

However, there are limitations to the study. First, it is limited to nurses’ experience. The research questions targeted the practice of primary care nurses. We recognize that input from patients’ and other health professionals’ experience is important and a study of their experience with integrated self-management support could help improve FMG nurses’ practice. Second, although recruitment strategies made it possible to target nurses with experience relevant to the phenomenon under investigation, many participants were nurses with several years of experience. In Quebec, nurses are unionized professionals and appointment to an FMG position is generally made through a combination of an interview and accumulated years of experience (seniority within an institution). Given that these are coveted positions, this type of participant profile is not surprising. This is not unique to this study and has been reported elsewhere [25, 86], but may have an impact on the transferability of results to other FMG nursing practices. Even if the nurses have a bachelor’s degree, integrated self-management support for people with CD and CMD remain complex, and nurses may need continuing education to ensure comprehensive care. Third, there may have been a social desirability bias and nurses may have given positive responses or overestimated their performance [87]. Only one data collection method was used and the use of additional methods could have allowed for triangulation of methods and different points of view [88]. However, given the wide geographical distribution, access to different settings across the province became complex and difficult. Although some authors note certain limitations associated with virtual interviews (e.g., confidentiality, technical difficulties), it facilitated this research and similar effectiveness and benefits to a face-to-face format have been demonstrated [89].

## Conclusion

Clinical integration of self-management support for CD and CMD is achieved through prevention (risk factors), health promotion (good lifestyle habits), and disease interaction education. A model of integrated self-management support in primary care nursing has been proposed to understand and implement future interventions, including the essential components of an integrated self-management support approach, as well as a detailed description of the support activities carried out by nurses. Still, there is a need for improvement in CMD self-management support, and gaps were identified, especially in terms of support for specific CMD (anxiety and depression). Possible solutions were discussed, including further development of technical and non-technical skills, especially regarding behaviour change therapies. Future integrated self-management support training should focus on CMD-related knowledge development, technical (e.g., behavioural change techniques) and non-technical skills (e.g., person-focused care approach and co-creation of self-management support process).

### Electronic supplementary material

Below is the link to the electronic supplementary material.

Supplementary Material 1

Supplementary Material 2

Supplementary Material 3

## Data Availability

The datasets used and/or analysed during the current study are available from the corresponding author on reasonable request.

## Abbreviations

CD: Chronic physical diseases
CMD: Common mental disorders
CoP: The Quebec FMG nurses’ virtual community of practice
FMG: Family medicine group
FMG-U: University family medicine group
QON: Quebec Order of Nurses
SLSJ: Saguenay-Lac-Saint-Jean
SMS: Self-management support
