# Supplementary material for: Integrated self-management support provided by primary care nurses to persons with chronic diseases and common mental disorders: a qualitative study
Source: BMC Prim Care. 2024 Jun 12;25:212. doi: 10.1186/s12875-024-02464-8 (PMC11167744; doi:10.1186/s12875-024-02464-8)
Supplement: Supplementary file 1 — Supplementary Material 1 [file 12875_2024_2464_MOESM1_ESM.docx]

**Additional file 1**

**Title:** Integrated self-management support provided by primary care nurses to persons with chronic diseases and common mental disorders: A qualitative study

**Interview questions based on research questions**

| **Research questions** | **Interview questions** |
| --- | --- |
| 1. How do nurses integrate SMS for people with physical CD and CMD at the clinical level? | In your interventions, how do you take into account both physical MC and TMC?  What strategies do you use in these situations, and why?  Do you have a particular approach with these people?  How do you involve the person in the care process?  How do you share responsibility for care? |
| 2. What SMS activities do nurses perform with people who have physical CD and CMD? | I'd like you to describe what it's like to support self-management in an encounter with a patient with CD and TMC.  What interventions do you use to support self-management of a person with both CD and TMC?  What tools do you use? |
