# Supplementary material for: Integrated self-management support provided by primary care nurses to persons with chronic diseases and common mental disorders: a qualitative study
Source: BMC Prim Care. 2024 Jun 12;25:212. doi: 10.1186/s12875-024-02464-8 (PMC11167744; doi:10.1186/s12875-024-02464-8)
Supplement: Supplementary file 2 — Supplementary Material 2 [file 12875_2024_2464_MOESM2_ESM.docx]

**Additional file 2**

**Title:** Integrated self-management support provided by primary care nurses to persons with chronic diseases and common mental disorders: A qualitative study

**Coding tree example of theme 1**

| **Subthemes and codes** | | **Theme** |
| --- | --- | --- |
| ***Person-focused approach***  **Adopt a holistic and human approach**   - Be a companion - Act as a guide - Treat her patients as if they were herself - "tender loving care" - "I'm not here for the doctor, but for the patient" - 100% in the present moment - Act as a mirror to the person - In the form of sharing - See the person as a whole   ***Adapting integrated SMS on an autonomy-dependency continuum***   - Prior understanding of the person - According to the needs of the person - According to their concerns - According to what the person wants   - Choices   - Priorities - According to their capabilities   - Preparation level   - Language adjustment to the patient   - Education level   - Literacy   - Limit - According to current state and progression - According to stages of change | ***Co-creation of SMS process through active participation and shared responsibility***   - Respect for decision-making autonomy - nurse helps person to be autonomous - strategies to make the person autonomous - clearly stated shared responsibility   - care contract   - in terms of follow-up   - in terms of treatment   - in terms of care procedures - benefits of shared responsibility - ensuring the dissipation of power in the relationship - challenges of shared responsibility - letting the person lead SMS - need for the person's participation   strategies to ensure active participation  **A nurse-person partnership relationship must be developed**   - Prioritize relationship building - benefits of developing a relationship - relationship-building techniques   **Assuring continuity of care and services**   - honoring relational continuity - ensure continuity of care - nurse acts as key player - liaison - openness to follow-up as needed | **Elements of the approach** |
