# Supplementary material for: Integrated self-management support provided by primary care nurses to persons with chronic diseases and common mental disorders: a qualitative study
Source: BMC Prim Care. 2024 Jun 12;25:212. doi: 10.1186/s12875-024-02464-8 (PMC11167744; doi:10.1186/s12875-024-02464-8)
Supplement: Supplementary file 3 — Supplementary Material 3 [file 12875_2024_2464_MOESM3_ESM.docx]

**Additional file 3**

**Title:** Integrated self-management support provided by primary care nurses to persons with chronic diseases and common mental disorders: A qualitative study

**Condensation of SMS activities based on the PRISMS taxonomy**

| **Integrated SMS activities** | **PRISMS taxonomy components** |
| --- | --- |
| Biopsychosocial therapeutic education | A1. Information about condition and /or its management  A7. Provision of equipment |
| Shared individualized action plan | A3. Provision of/agreement on specific clinical action plans and/or rescue medication  A12. Training/ rehearsal for psychological strategies (goal setting, action planning, problem-solving) |
| Biopsychosocial monitoring | A4. Regular clinical review  A5. Monitoring of condition with feedback |
| Adherence support | A6. Practical support with adherence (medication or behavioural) |
| Support for practical self-management skills | A9. Training/rehearsal to communicate with healthcare professionals  A10. Training/ rehearsal for everyday activities  A11. Training/ rehearsal for practical self-management activities |
| Lifestyle support | A14. Lifestyle advice and support |
| Psychological strategies | A12. Training/ rehearsal for psychological strategies |
| Social support and resource liaison | A2. Information about available resources  A13. Social support |
| SMS accessibility measures | A8. Provision of easy access to advice or support when needed |
